# Serum biomarkers in patients with drug-resistant epilepsy: a proteomics-based analysis

**DOI:** 10.3389/fneur.2024.1383023

**Published:** 2024-03-22

**Authors:** Mian Ma, Ying Cheng, Xiaoxia Hou, Zhisen Li, Meixia Wang, Bodun Ma, Qingzhang Cheng, Zhiliang Ding, Hongxuan Feng

**Affiliations:** ^1^Department of Neurosurgery, The Affiliated Suzhou Hospital of Nanjing Medical University, Suzhou Municipal Hospital, Suzhou, Jiangsu, China; ^2^Suzhou Jinchang Street Bailian Community Health Service Center, The Affiliated Suzhou Hospital of Nanjing Medical University, Suzhou Municipal Hospital, Suzhou, Jiangsu, China; ^3^Department of Neurology, The Affiliated Suzhou Hospital of Nanjing Medical University, Suzhou Municipal Hospital, Suzhou, Jiangsu, China; ^4^Department of Radiology, The Affiliated Suzhou Hospital of Nanjing Medical University, Suzhou Municipal Hospital, Suzhou, Jiangsu, China

**Keywords:** drug-resistant epilepsy, proteomics, biomarkers, serum, antiepileptic drugs

## Abstract

**Objective:**

To investigate the serum biomarkers in patients with drug-resistant epilepsy (DRE).

**Methods:**

A total of 9 DRE patients and 9 controls were enrolled. Serum from DRE patients was prospectively collected and analyzed for potential serum biomarkers using TMT18-labeled proteomics. After fine quality control, bioinformatics analysis was conducted to find differentially expressed proteins. Pathway enrichment analysis identified some biological features shared by differential proteins. Protein–protein interaction (PPI) network analysis was further performed to discover the core proteins.

**Results:**

A total of 117 serum differential proteins were found in our study, of which 44 were revised upwards and 73 downwards. The up-regulated proteins mainly include UGGT2, PDIA4, SEMG1, KIAA1191, CCT7 etc. and the down-regulated proteins mainly include ROR1, NIF3L1, ITIH4, CFP, COL11A2 etc. Pathway enrichment analysis identified that the upregulated proteins were mainly enriched in processes such as immune response, extracellular exosome, serine-type endopeptidase activity and complement and coagulation cascades, and the down-regulated proteins were enriched in signal transduction, extracellular exosome, zinc/calcium ion binding and metabolic pathways. PPI network analysis revealed that the core proteins nodes include PRDX6, CAT, PRDX2, SOD1, PARK7, GSR, TXN, ANXA1, HINT1, and S100A8 etc.

**Conclusion:**

The discovery of these differential proteins enriched our understanding of serum biomarkers in patients with DRE and potentially provides guidance for future targeted therapy.

## 1 Introduction

Epilepsy is a chronic recurrent brain disease resulted from highly synchronized abnormal discharges of neurons in the brain, primarily manifested by sudden onset of limb convulsions, muscle tonus, sensory abnormalities, and loss of consciousness (1). It is estimated that there are about 70 million epilepsy patients worldwide, and the prevalence rate in China is 4.6‰~7.0‰, with an annual growth of (28.8~35.0)/100,000 (2, 3). The etiology of epilepsy has been reported to be related to cortical developmental disorders, central nervous system infections, tumors, trauma, cerebrovascular diseases, neurodegeneration, and genetic metabolic diseases (1, 4–7). Several brain regions are involved in seizures including, thalamus, hippocampus, amygdala, insula, and anterior cingulate gyrus.

For a long time, the main clinical practice for seizure control has been the use of one or more antiepileptic drugs (AEDs). These drugs inhibit focal neuronal overdischarge through a variety of pharmacological mechanisms, including modulation of voltage-gated ion channels, enhancement of GABAergic activity, inhibition of glutamatergic processes, and alteration of neurotransmitter release (7, 8). Despite the many choices of AEDs, about 30% of patients applying two or more AEDs still have uncontrolled seizures, which is called medically DRE (9–14). The frequency of seizures greatly impacts the patient’s quality of life, and in severe cases, may even jeopardize the patient’s life (15).

The causes of DRE include congenital factors, rapid drug metabolism and functional resistance, among which the expression of drug resistance genes and the production of multi-drug resistance proteins play an important role (16). Therefore, there is an urgent need to develop accessible protein biomarkers that can help predict disease progression and treatment response, which is thought to help reveal the mechanisms of both epileptogenesis and drug resistance (17, 18). Blood, as one of the most clinically accessible body fluids, has great potential to generate accessible biomarkers for epilepsy (19). With the popularity of protein quantification techniques, the identification of blood markers for various diseases has been greatly facilitated. In this study, we collected serum from DRE patients and took quantitative proteomic analysis based on TMT18 isotope labeling with the aim of identifying new biomarkers to provide diagnosis and treatment basis for DRE patients.

## 2 Materials and methods

Clinical data and serum samples of nine patients with DRE and nine controls without seizures who attended the Department of Neurology at the Affiliated Suzhou Hospital of Nanjing Medical University from January 2021 to August 2023 were included and analyzed in this study. All patients gave informed consent and authorized signatures. This study was approved by the Ethics Committee of the Affiliated Suzhou Hospital of Nanjing Medical University.

### 2.1 Clinical data collection

General information and medical history of the patients were collected, including gender, age, diagnosis, age of onset, seizure form, medication history and seizure frequency. Inclusion Criteria: Compliance with the diagnostic criteria for DRE as revised by the International League Against Epilepsy (ILAE) in 2010 (13). Exclusion Criteria: Secondary seizures due to other diseases such as cerebrovascular disease, traumatic brain injury and encephalitis.

### 2.2 Proteomic profiling in human serum

TMT18 labeling based proteomic analysis was performed to investigate the changes in serum proteomics of DRE patients.

#### 2.2.1 High abundance protein removal

Serum was centrifuged at 3,000 g for 10 min then 10ul of the supernatant was aspirated to remove the TOP14 high abundance protein according to the instructions of High-Select ™ Top14 Abundant Protein Depletion Resin (Catalog Numbers: A36370), and the 10kD filter membrane was used to concentrate and extract the protein by utilizing urea lysate. The serum after removing the high abundance was concentrated and protein extracted using 10kD filter membrane and urea lysate.

#### 2.2.2 Determination of protein concentration by Bradford method

(1) Dilute the BSA standard solution (5 mg/mL) with 8 M lysate or water to 0.5 mg/mL as the application solution, i.e., 0.5 μg/μL; (2) Add the application solution at 0, 1, 2, 4, 8, 12, 16, 20 μL into the standard wells of the enzyme plate, and add lysis solution to 20 μL; (3) Add 1 μL of sample into the sample well of the enzyme plate and make up to 20 μL with lysate; (4) Add 200 μL of G250 staining solution to each well and leave for 3–5 min at room temperature; (5) Determine the standard curve and protein concentration by enzyme labeling, take 20ug of protein from each sample for TMT quantitative proteomics analysis, and 4ug for SDS-PAGE to observe the removal of high abundance proteins.

#### 2.2.3 Proteolytic cleavage

(1) Add 1 M DTT to its final concentration of 5 mM, 56°C water bath for 30 min; (2) The sample was reduced to room temperature, 1 M IAA was added to its final concentration of 14 mM with 30 min of operation away from light; (3) Add 1 M DTT to a final concentration of 5 mM with 15 min of operation away from light; (4) Dilute the concentration of urea by adding 25 mM Tris–HCl pH 8.2; (5) Add 0.1 M CaCl2 to its final concentration of 1 mM; (6) Add Trypsin to a concentration of 10 ng/μl; (7) 37°C, overnight.

#### 2.2.4 Peptide purification

(1) Add TFA to terminate the enzymatic cleavage of Trypsin; (2) Centrifuge for 10 min 15,000 g; (3) Desalting of the column (Waters) with 10 mg C18; (4) Liquid: Liquid A (0.1% TFA); Liquid B (50% ACN + 0.5% HAc); (5) 1 mL 100% ACN to rinse the desalting column; (6) 1 mL of liquid B to flush the desalination column; (7) 1 mL of liquid A to equilibrate the desalting column; (8) Sampling 2 times; (9) 1 mL of liquid A to wash the salt; (10) 1 mL of liquid B elution, save the eluent and pump dry.

#### 2.2.5 TMT labeling

(1) TMT18 labeling reagent was placed at room temperature, dissolved in anhydrous acetonitrile and incubated for 5 min; (2) Add the labeling reagent to each TEAB-dissolved sample correspondingly; (3) Reaction at room temperature for 1 h; (4) 5% hydroxylamine suspension; (5) Mix proportionally and lyophilize.

#### 2.2.6 HPRP isolation

Chromatograph: Waters H-class; Chromatographic column: Waters BEH C18 Column, 1.7 μm, 1 mm × 150 mm; Mobile phase: A (20 mM ammonium formate, pH = 10), B (100%ACN).

#### 2.2.7 LC-MS/MS analysis

(1) Chromatographic conditions: Chromatograph: EASY nLC 1,200 chromatograph (ThermoFisher); Mobile phase: A (water/0.1%FA), B (80%ACN/0.1%FA); Analytical column: 75 μm × 15 cm, Inhouse packed C18 column, 1.9 μm; Flow rate: 0.3 μL/min. (2) Mass spectrometry conditions: Mass spectrometer: Orbitrap Fusion Lumos high-resolution mass spectrometer (ThermoFisher). Software setup parameters: spray voltage, 2.2 kV; parent ion scanning was detected by Orbitrap, mass scanning range: 350–1,500, resolution set to 60 K; daughter ions were detected by Orbitrap, resolution set to 50 K, HCD collision mode, energy 36%.

### 2.3 Bioinformatics analysis

Principal Component Analysis (PCA) is resulted from multidimensional statistical analysis, which can be used to visualize the degree of aggregation and dispersion of the samples through PCA score plots. FactoMine and ggplot2 R packages were used to perform PCA analysis and visualization. Volcano Plot, a scatter plot representing differential expression of features, was plotted in this study based on the Complexheatmap R package. A threshold of *p*-value < 0.05, abs (fold change) ≥ 1.2 was used to characterize the direction of protein change. Correlation of protein expression between samples was calculated by calculating the Pearson correlation coefficient and visualized using the pheatmap R package. Gene Ontology (GO) is a crucial bioinformatics analysis method by characterizing various attributes of genes and gene products. GO explains the biological roles of proteins from three different perspectives: biological process (BP), cellular component (CC) and molecular function (MF). Kyoto Encyclopedia of Genes and Genomes (KEGG) annotates the information of identified proteins at the level of biological pathways. R packages ClusterProfiler and AnnotationHub were used for the above functional annotations, respectively. The online STRING tool^1^ was used to analyze PPI network.

## 3 Results

### 3.1 General statistics

All 9 patients with DRE had uncontrolled seizures despite taking 2–3 AEDs in the previous 1 year. Four of them were males and 5 were females with a mean (50.11 ± 11.3) years of age; three of them had a seizure frequency of 1–3 times/month, four of them had 3–5 times/month, and two of them had more than 5 times/month. Nine control patients were other hospitalized patients without seizures. There were 5 males and 4 females with a mean of (47.89 ± 12.83) years. For details, see Table 1.

**Table 1:** Patient clinical information used for this study.

Patient	Gender	Age	Diagnosis	Age of onset	Seizure form	AEDs medication	Seizure frequency
Norm_1	Male	56	Normal	/	/	/	/
Norm_2	Female	34	Normal	/	/	/	/
Norm_3	Male	25	Normal	/	/	/	/
Norm_4	Female	45	Normal	/	/	/	/
Norm_5	Male	46	Normal	/	/	/	/
Norm_6	Female	57	Normal	/	/	/	/
Norm_7	Male	45	Normal	/	/	/	/
Norm_8	Female	56	Normal	/	/	/	/
Norm_9	Male	67	Normal	/	/	/	/
	Male: 55.56%	47.89 ± 12.83					
DRE_1	Female	46	Drug-resistant epilepsy	41	Complex partial seizure	Topiramate and Carbamazepine	1–3/months
DRE _2	Male	37	Drug-resistant epilepsy	32	Generalized tonic–clonic seizures	Levetiracetam and Sodium Valproate	1–3/months
DRE _3	Female	46	Drug-resistant epilepsy	25	Simple partial seizures	Topiramate and Carbamazepine	3–5/months
DRE _4	Male	46	Drug-resistant epilepsy	38	Complex partial seizure	Levetiracetam and Sodium Valproate	3–5/months
DRE _5	Female	56	Drug-resistant epilepsy	44	Generalized tonic–clonic seizures	Levetiracetam and Sodium Valproate	3–5/months
DRE _6	Male	77	Drug-resistant epilepsy	56	Simple partial seizures	Topiramate and Pirenpanet	1–3/months
DRE _7	Female	46	Drug-resistant epilepsy	42	Complex partial seizure	Levetiracetam and Sodium Valproate	>5/months
DRE _8	Male	45	Drug-resistant epilepsy	41	Generalized tonic–clonic seizures	Levetiracetam and Sodium Valproate	3–5/months
DRE _9	Female	52	Drug-resistant epilepsy	48	Simple partial seizures	Pirenpanet and Carbamazepine	>5/months
	Male: 44.44%	50.11 ± 11.33		40.78 ± 8.87	Complex partial seizure: 33.33%; Generalized tonic–clonic seizures: 33.33%; Simple partial seizures: 33.33%		1–3/months: 33.33%; 3–5/months: 44.44%; >5/months: 22.22%

### 3.2 Data distribution was comparable between groups

**Figure 1:**
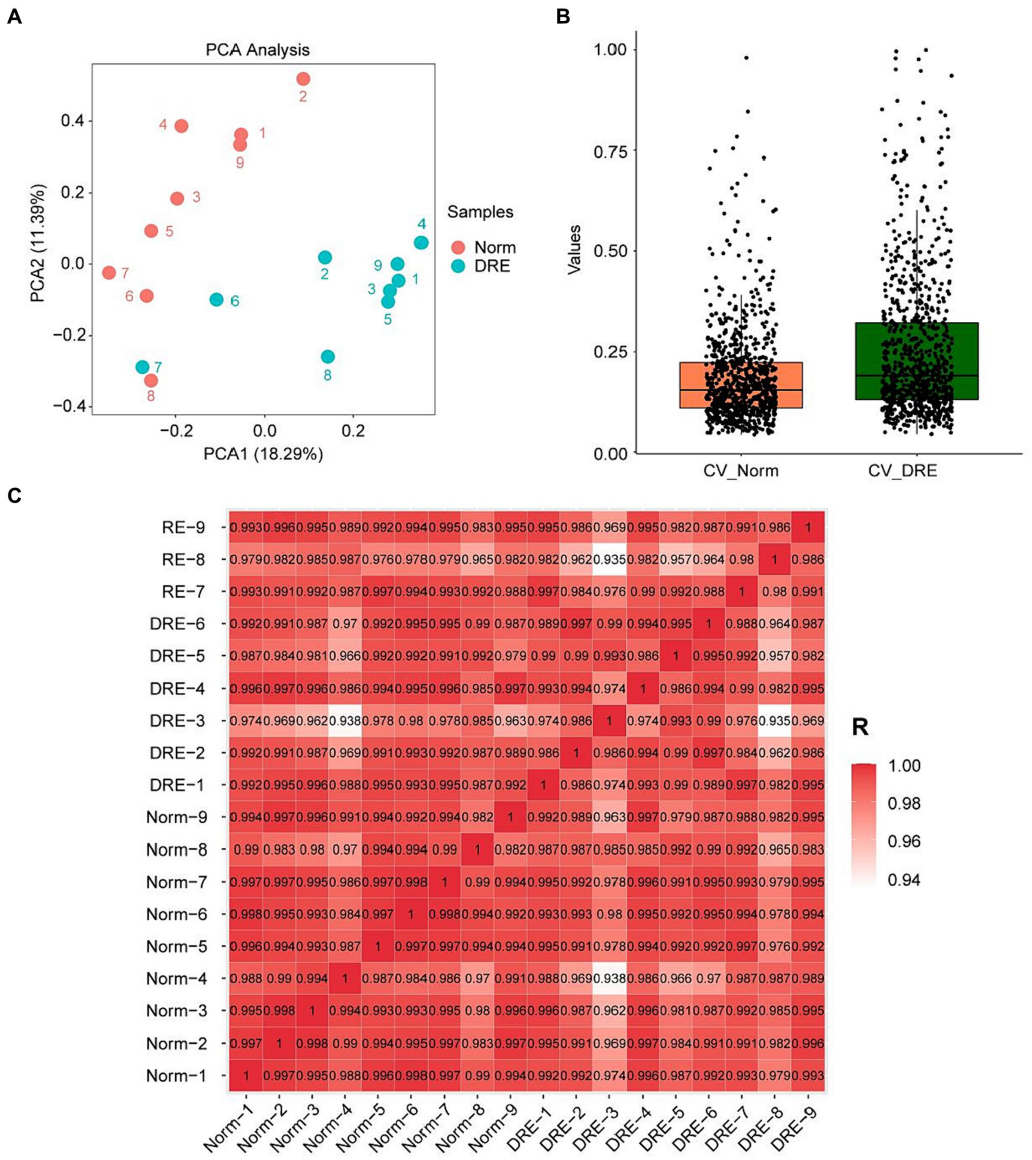
Characterization of the DRE and control group data indicating good quality control. **(A)** PCA visualization of the data distribution of the two groups. **(B)** Comparison of the coefficient of variation of the data distribution. **(C)** Correlation analysis of the serum proteins between samples based on the Pearson’s coefficient.

Before formally analyzing the data, we first performed a series of pre-analyses on the distribution of the data to verify if the quality control was satisfactory. PCA manifested that samples from the normal and DRE groups were clustered separately, with similar data characteristics within groups and significant distinctions between groups (Figure 1A). We next quantified the Coefficient of Variance (CV) of the data distribution of the two groups (Figure 1B). Our results suggested that the median CV of both groups did not exceed 0.2, suggesting a lower degree of dispersion and a higher degree of reproducibility. Finally, we visualized the correlation between these samples based on the Pearson correlation coefficient (Figure 1C). All the correlation coefficients within the normal group were larger than 0.97, with less heterogeneity ensuring reliability as a control group. In contrast, the correlation coefficients within the DRE group ranged from 0.93 to 1, indicating some heterogeneity among these DRE patients compared with the normal control. This is in line with our expectations, as numerous factors, such as epileptogenic foci, differences in seizure form and frequency, and medication administration, may cause serum proteomic variability in these patients. Overall, these data suggest that the serum proteomic data collected from both groups of patients were of good quality control and can be used for further data analysis.

### 3.3 Analysis of serum proteins differences between groups

**Figure 2:**
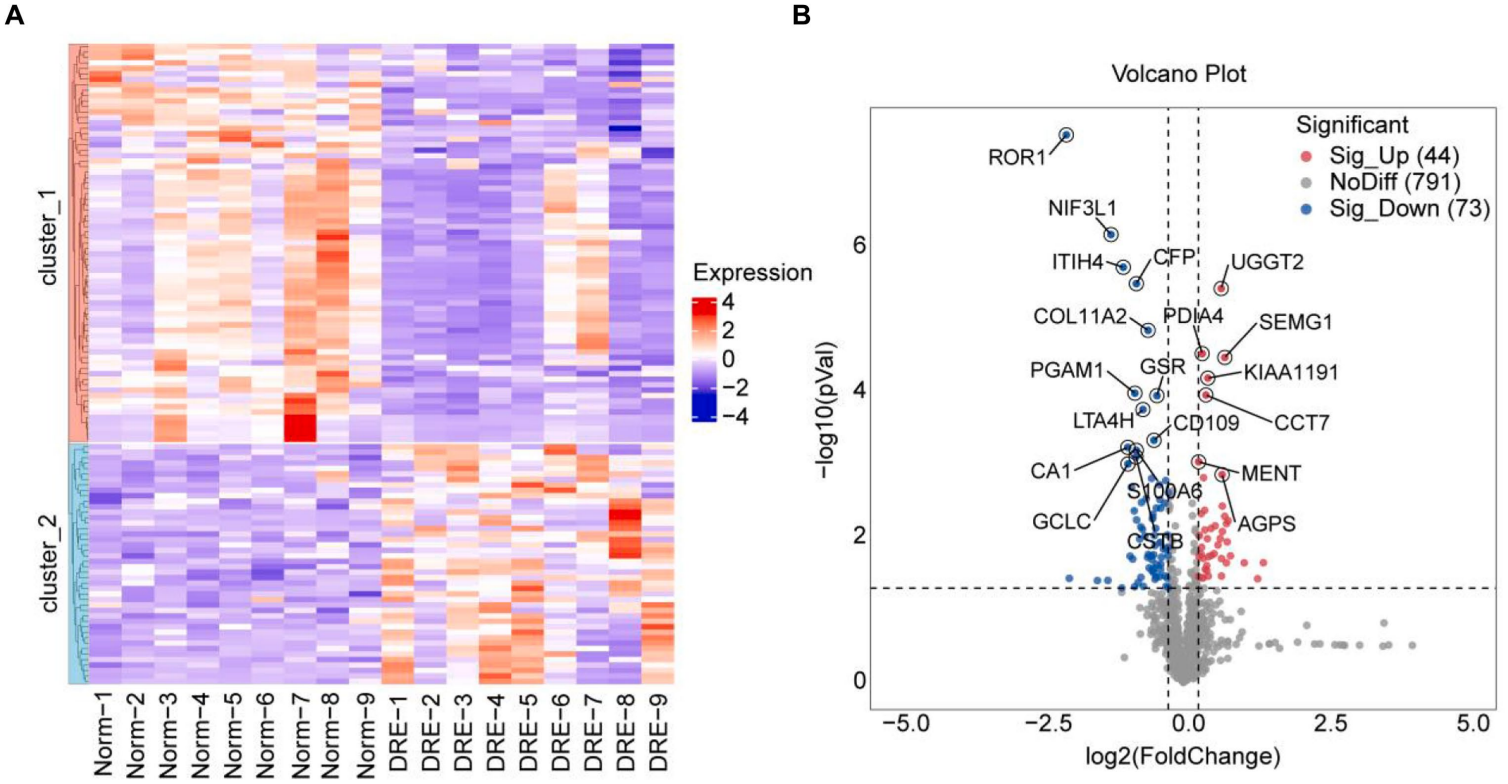
Difference analysis of serum proteins between the two groups. **(A)** Heatmap visualization of differential proteins between the two groups. **(B)** Volcano plot of differential proteins between the two groups. Use *p*-value < 0.05, fold change ≥ 1.2 as the threshold.

Next, we performed a differential analysis of serum proteins among the two groups. Totally, 908 proteins were detected by quantitative analysis of TMT18 labeling. The differences in serum secreted proteins between the control and DRE groups were quite dramatic. As shown in the heatmap, 117 differential proteins were detected using *p*-value < 0.05, fold change ≥ 1.2 as the threshold (Figure 2A). Compared with the normal group, 44 and 73 proteins were up-regulated and down-regulated in the serum of DRE patients (Figure 2B). Detailed differential protein profiles are shown in Supplementary Table 1. The data presented in the study are deposited in the ProteomeXchange Consortium^2^ via the iProX partner repository (20, 21), accession number PXD050309.

### 3.4 Functional enrichment analysis of differentially expressed proteins

**Figure 3:**
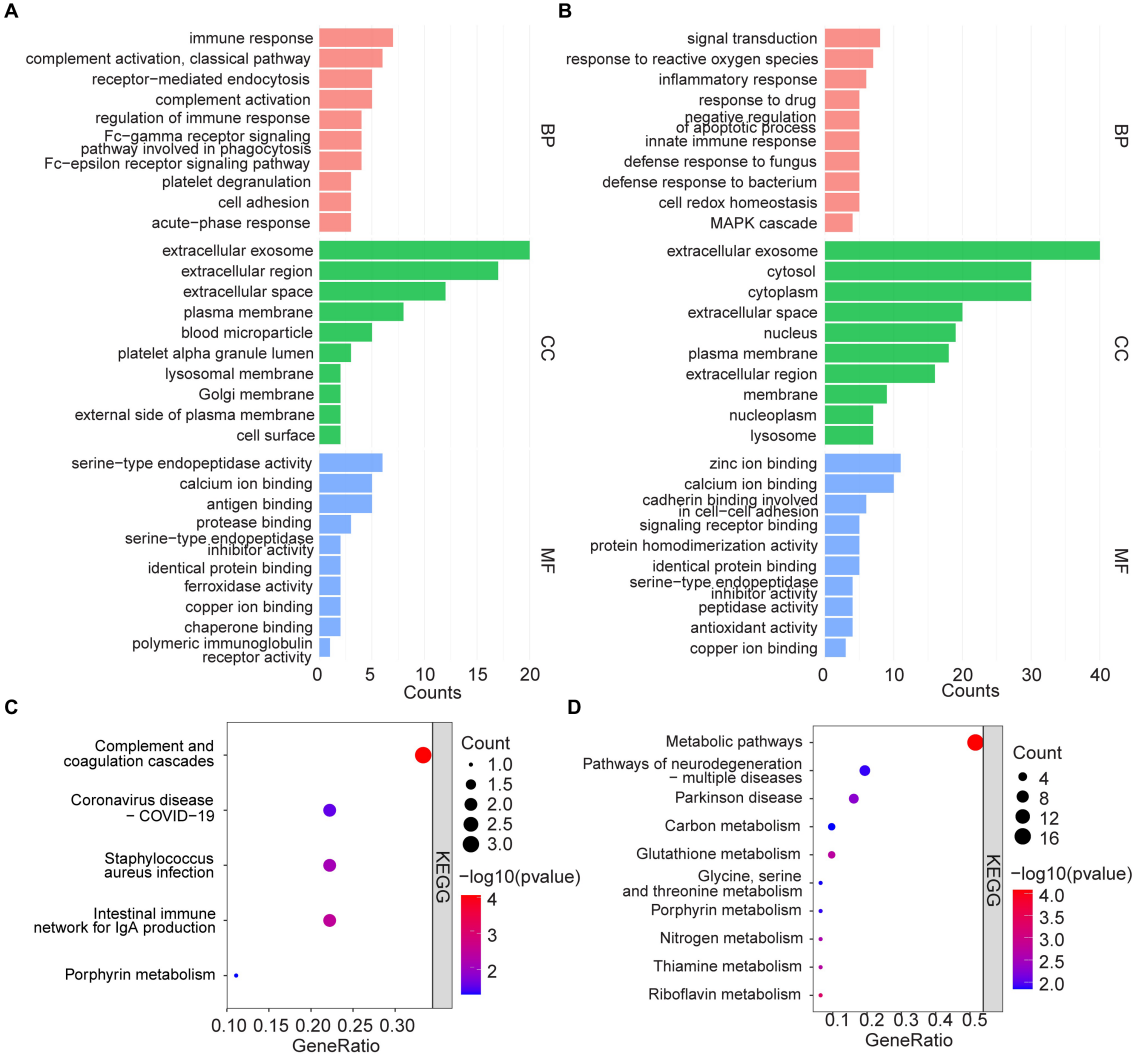
GO analysis and KEGG pathway enrichment analysis. **(A,C)** GO and KEGG enrichment analysis of upregulated serum proteins. **(B,D)** GO and KEGG enrichment analysis of down-regulated serum proteins.

Functional enrichment of 44 high-expressed proteins and 73 low-expressed proteins were analyzed to identify the corresponding biological characteristics based on GO and KEGG. GO enrichment analysis revealed that upregulated proteins were enriched in BP such as immune response, complement activation, and receptor-mediated endocytosis; CC such as extracellular exosome, extracellular regions and spaces, plasma membranes, and blood microparticles; as well as MF such as serine-type endopeptidase activity, and binding of calcium ions, antigens, and proteases (Figure 3A). KEGG pathway analysis suggested that up-regulated proteins were enriched in pathways such as complement and coagulation cascades, coronavirus disease-COVID-19, *Staphylococcus aureus* infection, and intestinal immune network for IgA production (Figure 3C).

In contrast, down-regulated proteins were enriched in GO analysis for BP such as signal transduction, response to reactive oxygen species and inflammation, response to drugs, and negative regulation of apoptotic processes; CC such as extracellular exosome, cytosol, cytoplasm, and extracellular space; as well as MF such as zinc/calcium ion binding, cadherin binding related to cell–cell adhesion, and signaling receptor binding (Figure 3B). KEGG analysis, on the other hand, suggested that the down-regulated proteins were enriched in metabolic pathways, multiple neurodegeneration, and other pathways (Figure 3D).

### 3.5 Protein interaction network analysis

**Figure 4:**
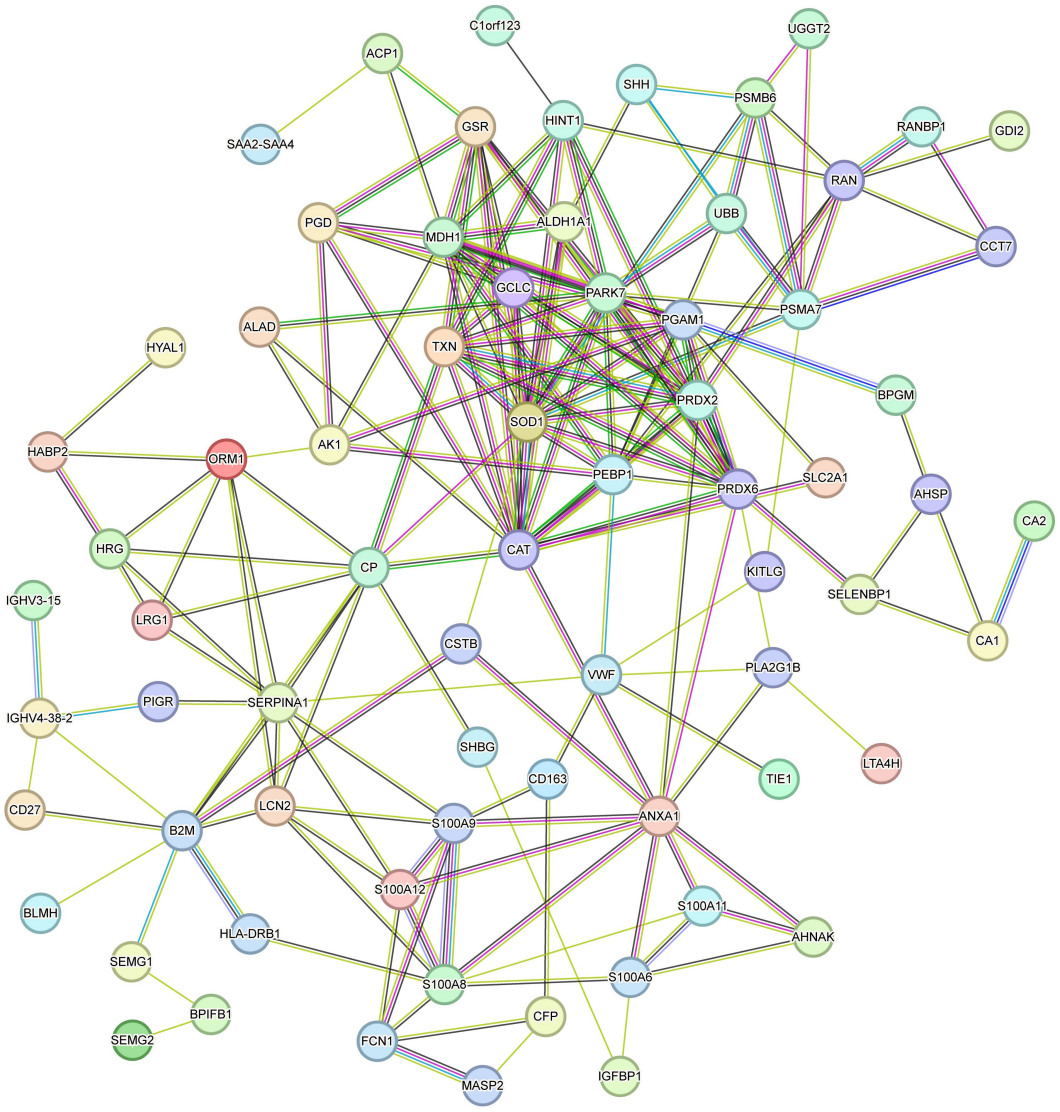
PPI network analysis of differential proteins.

With GO and KEGG enrichment analyses, we gained a general understanding of the classification of differentially expressed proteins. To further understand the interactions between differentially expressed proteins, a PPI network analysis was performed using the STRING tool (Figure 4). There were 69 nodes in total. The top 10 highest ranked nodes were, in order, PRDX6, CAT, PRDX2, SOD1, PARK7, GSR, TXN, ANXA1, HINT1, and S100A8. We found that most of these differential proteins had interactions, which were evident at some of the junctional confluences. The central roles of these hub proteins in the network foreshadowed their deep involvement in the deep involvement in complex protein interactions.

## 4 Discussion

By analyzing the above data and reviewing the published literature, we found much evidence consistent with the results of this study. Of the 117 serum differential proteins we identified, many have been corroborated in epilepsy patients and animal models.

S100A6 (calcineurin) is an EF-hand calcium-binding protein that is thought to have multiple functions, including calcium homeostasis and neuronal degeneration (22). One study based on an epileptic rat model showed that S100A6 protein levels were significantly elevated in the cortex and hippocampal CA1 region on day 14 after amygdala stimulation (23). Further findings suggest that elevated S100A6 immunoreactivity is associated with astrocyte proliferation. This is in contrast to our findings in human serum, as our sequencing results indicate that S100A6 is decreased in the serum of patients with DRE. The decrease in S100A6 protein may be involved in the abnormalities of neuronal excitability in part through dysregulation of calcium homeostasis. This inconsistency may be due to a number of different factors such as tissue samples, assay methods, and so on. Nonetheless, this evidence points to the involvement of S100A6 in seizures.

The glutamate cysteine ligase catalytic subunit (GCLC) is also one of the down-regulated proteins in serum from patients with DRE in this study. As we all known, oxidative stress plays a crucial part in neuronal damage and gliosis. It has been suggested that phenytoin sodium, an antiepileptic drug, may have an effect on GCLC. Upregulation of GCLC by phenytoin may increase hippocampal glutathione (GSH) levels and protect hippocampal cells from oxidative stress damage, which in turn mediates its potential antiepileptic effects (24).

Progressive myoclonus epilepsy of the Unverricht-Lundborg type (EPM1) is autosomal recessive, 6–13 years of age onset, manifested by spontaneous myoclonic and generalized tonic–clonic seizures more sensitive to stimulation, with the development of the disease in the later stages of the disease may have ataxia, dysarthria and dementia (25). Evidence suggested that EPM1 is caused by genemutations in the cystatin B (CSTB) (26). Accordingly, the CSTB-deficient mouse model contains the main features of EPM1, including myoclonic seizures (27, 28). In the present study, CSTB was significantly reduced in the serum of DRE patients, suggesting its potential as a DRE-related biomarker.

PRDX6 was identified as differentially expressed in epileptic brain tissues from patients with childhood cortical dysplasia (29). In addition, PRDX6 was reported to be upregulated in CA1 astrocytes from chronically epileptic rats. Activation of specificity protein 1 (Sp1) was thought to mediate this increase and thus leading to prolonged seizure activity through autophagic astrocyte degeneration (30). The team’s work 1 year later further suggested that aiPLA2 activity of PRDX6 in astrocytes may be one of the upstream influencing factors of seizure duration in the epileptic hippocampus (31). In our data, serum levels of PRDX6 were notably decreased in patients with DRE compared with controls, suggesting the heterogeneity of different types of epilepsy disorders in different species.

Several proteomic-based analyses have suggested that superoxide dismutase 1 (SOD1) is notably reduced in the cerebrospinal fluid (CSF) of epilepstic patients (32, 33). Further work by Chen et al. suggests that SOD1 levels are reduced in the CSF of epileptic patients, especially those with DRE. Low levels of CSF-SOD1 may be a predictor of resistance to AEDs in epilepstic patients (33). SOD1 may be secreted into both the CSF and the blood circulation, as suggested by our results.

Hippocampal sclerosis (HS) is a common histopathologic anomaly in Mesial temporal lobe epilepsy (MTLE) (34). PARK7 encodes DJ-1, a mutation of which is a rare reason of early-onset recessive Parkinson’s disease (35, 36). Parkinson protein 7 (PARK7) has been reported to be down-regulated in hippocampal tissues of MTLE patients (37) as well as in DG tissues of epileptic rats (34), which is consistent with our proteomic analysis results.

Inflammatory processes have been described as key mechanisms in the pathophysiology of temporal lobe epilepsy (TLE). The anti-inflammatory endogenous protein membrane-associated protein A1 (ANXA1) is an interesting target involved in the progression of many neurological diseases, modulating neuroinflammation by inhibiting leukocyte migration and pro-inflammatory mediator release (38). A 2015 study demonstrated that ANXA1, by attenuating inflammatory damage and protecting neurons plays an important role in TLE (39). Besides, another study also revealed a beneficial role of ANXA1 in epilepsy (40). The anti-inflammatory glucocorticoid receptor (GR)-ANXA1 pathway is defective during experimental seizure progression. Administration of exogenous ANXA1 reduced the duration of seizure activity to some extent. Our results also suggest that ANXA1 is decreased in the serum of DRE patients. The above results indicate that targeted upregulation of ANXA1 may be able to exert neuroprotective effects and alleviate seizure progression by enhancing anti-inflammation.

Surprisingly, we also identified many differential proteins such as UGGT2, SEMG1, PDIA4, ROR1, NIF3L1, and ITIH4, that have not previously been directly reported with epilepsy. UDP-glucose/glycoprotein glucosyltransferase 2 (UGGT2), a protein associated with the recognition and modification of misfolded proteins (41), was significantly increased in our study. Recently, endoplasmic reticulum (ER) stress in response to the accumulation of intracellular misfolded proteins has been identified as a key etiological factor contributing to epilepsy-induced neuronal damage (42). This evidence implies a neuronal response to injury after seizures. ROR1 is a member of the ROR subfamily of tyrosine kinase receptors (RTKs) that interacts with Wnt-5a to mediate its effects on synaptogenesis (43). Down-regulation of ROR1 resulted in a significant reduction of synapse formation in cultured hippocampal neurons (44). Our results suggest that serum ROR1 levels are lower in patients with DRE. There is evidence that the reduction of inhibitory synapses in GABAergic neurons could be involved in the hyperexcitability of cortical neurons (45). Whether ROR1 reduction is involved in the development of epilepsy through inhibitory synaptic remodeling needs to be demonstrated in further experiments.

In summary, the reliability of our data has been fully confirmed by existing studies. The identification of these differentially regulated proteins not only points the way to the search for potential biomarkers in DRE patients, but also provides guidance for targeted precision therapy in these patients. Until then, a large number of extensive and in-depth studies are urgently needed to further elucidate the involvement of these proteins in epilepsy pathology.

There are also inevitably some limitations to our study. First, limited by the sample size, our conclusions need to be further validated in a larger cohort. Second, this study only identified proteins in serum samples, which unavoidably confounds the many proteins secreted from other organs. Obviously, physically closer and purer to the epileptic foci are the adjacent brain tissues and CSF, which is a direction for further research. Finally, thanks to the rapid advances in multi-omics sequencing in recent years, single-cell proteomic analyses can quantify protein levels at the level of individual cells. The next step should be to combine multi-omics analysis including genomics, transcriptomics, proteomics and metabolomics to elucidate the pathological process of epilepsy in depth from multiple dimensions.

## 5 Conclusion

Our study used TMT18-labeled proteomics to analyze serum protein expression in 9 DRE patients vs. controls, and identified a number of differential proteins with potential to be used as serum markers, some of which have been reported in previous studies and some of which are proposed by us for the first time. These differential proteins are expected to serve as biomarkers and potential therapeutic targets in the future.

## Data availability statement

The original contributions presented in the study are publicly available. This data can be found at: https://www.iprox.cn//page/project.html?id=IPX0008305000.

## Ethics statement

The studies involving humans were approved by the Ethics Committee of the Affiliated Suzhou Hospital of Nanjing Medical University. The studies were conducted in accordance with the local legislation and institutional requirements. The participants provided their written informed consent to participate in this study.

## Author contributions

MM: Investigation, Writing – original draft. YC: Project administration, Writing – original draft. XH: Funding acquisition, Writing – review & editing. ZL: Methodology, Software, Writing – review & editing. MW: Data curation, Formal analysis, Writing – original draft. BM: Formal analysis, Writing – review & editing. QC: Data curation, Resources, Writing – review & editing. ZD: Funding acquisition, Supervision, Writing – review & editing. HF: Funding acquisition, Resources, Writing – review & editing.

## Footnotes

^1^ https://cn.string-db.org/

^2^ https://proteomecentral.proteomexchange.org
