# Supplementary material for: Serum biomarkers in patients with drug-resistant epilepsy: a proteomics-based analysis
Source: Front Neurol. 2024 Mar 22;15:1383023. doi: 10.3389/fneur.2024.1383023 (PMC10995353; doi:10.3389/fneur.2024.1383023)
Supplement: Supplementary file 1 [file Table_1.docx]

**Supplementary Table 1. Summary Table of Differential Proteins.**

| **Protein.ID** | **Gene names** | **Norm_mean** | **Epilepsy_mean** | **FC(E/N)** | **p_value** | **Diff** |
| --- | --- | --- | --- | --- | --- | --- |
| Q01973 | ROR1;ROR2 | 955666.6667 | 230023.3333 | 0.240694 | 2.84E-08 | Down |
| P80511 | S100A12 | 2427434.444 | 606158.8889 | 0.249712 | 0.036755 | Down |
| P05109 | S100A8 | 13631955.56 | 4787966.667 | 0.351231 | 0.039343 | Down |
| P04083 | ANXA1 | 662682.2222 | 264386.6667 | 0.398964 | 0.039169 | Down |
| Q9GZT8 | NIF3L1 | 183066.6667 | 75867.55556 | 0.414426 | 6.72E-07 | Down |
| P06702 | S100A9 | 5941600 | 2808866.667 | 0.472746 | 0.049846 | Down |
| H7C0L5 | ITIH4 | 5276422.222 | 2539800 | 0.481349 | 1.9E-06 | Down |
| P00915 | CA1 | 13543622.22 | 6887211.111 | 0.508521 | 0.000577 | Down |
| D6RGF8 | GCLC | 498541.1111 | 254051.1111 | 0.509589 | 0.000976 | Down |
| P31949 | S100A11 | 157468.2222 | 82334.77778 | 0.522866 | 0.018153 | Down |
| P32119 | PRDX2 | 17184466.67 | 9130777.778 | 0.531339 | 0.002044 | Down |
| P00441 | SOD1 | 4981622.222 | 2689285.556 | 0.539841 | 0.019955 | Down |
| P00918 | CA2 | 8957033.333 | 4921833.333 | 0.549494 | 0.004318 | Down |
| Q9NZD4 | AHSP | 373577.7778 | 206333.5556 | 0.552318 | 0.048061 | Down |
| P18669 | PGAM1;PGAM4;PGAM2 | 364124.4444 | 201821.1111 | 0.554264 | 0.000103 | Down |
| P04080 | CSTB | 35939.11111 | 20184.55556 | 0.561632 | 0.000782 | Down |
| R4GN98 | S100A6 | 1318175.556 | 742994.4444 | 0.563654 | 0.000633 | Down |
| P27918 | CFP | 35664000 | 20118777.78 | 0.56412 | 3.19E-06 | Down |
| H3BPK3 | HAGH | 360220 | 203781.1111 | 0.565713 | 0.005763 | Down |
| D6RC06 | HINT1 | 938504.4444 | 540485.5556 | 0.575901 | 0.041959 | Down |
| C9JLK2 | APEH | 126243.3333 | 74141.88889 | 0.587293 | 0.010521 | Down |
| P00568 | AK1 | 732230 | 436188.8889 | 0.595699 | 0.007177 | Down |
| P30041 | PRDX6 | 1140374.444 | 680354.4444 | 0.596606 | 0.035587 | Down |
| P04040 | CAT | 6784333.333 | 4074333.333 | 0.60055 | 0.003317 | Down |
| C9JXG8 | RANBP1 | 125866.5556 | 76435.66667 | 0.607275 | 0.007739 | Down |
| P09960 | LTA4H | 2570944.444 | 1571922.222 | 0.611418 | 0.000174 | Down |
| Q99497 | PARK7 | 1636930 | 1006285.556 | 0.61474 | 0.047364 | Down |
| P00352 | ALDH1A1 | 1128153.333 | 707020 | 0.626706 | 0.009609 | Down |
| A0A3B3IU56 | HMBS | 77784.88889 | 49480 | 0.636113 | 0.002933 | Down |
| H7BZT7 | ESD | 178886.6667 | 115726.2222 | 0.646925 | 0.018835 | Down |
| A0A0G2JHD9 | COL11A2 | 231051.1111 | 150407.7778 | 0.650972 | 1.4E-05 | Down |
| P07384 | CAPN1 | 476103.3333 | 310687.7778 | 0.652564 | 0.017207 | Down |
| P52209 | PGD | 956866.6667 | 630905.5556 | 0.659345 | 0.02638 | Down |
| P81605 | DCD | 6047100 | 4090055.556 | 0.676366 | 0.017959 | Down |
| X6R8F3 | LCN2 | 1343221.111 | 912607.7778 | 0.679417 | 0.001543 | Down |
| P10599 | TXN | 957892.2222 | 652745.5556 | 0.681439 | 0.028859 | Down |
| F8W062 | PLA2G1B | 216612.2222 | 148018.2222 | 0.683333 | 0.020376 | Down |
| B5MDF5 | RAN | 347527.7778 | 238354.4444 | 0.685857 | 0.005551 | Down |
| P07738 | BPGM | 1849500 | 1272135.556 | 0.687827 | 0.005333 | Down |
| J3QS39 | UBB;RPS27A;UBC;UBA52 | 87857.66667 | 60541.77778 | 0.689089 | 0.004849 | Down |
| A0A0A0MSI0 | PRDX1 | 377108.8889 | 262662.2222 | 0.696516 | 0.017393 | Down |
| B8ZZ51 | MDH1 | 526698.8889 | 366866.6667 | 0.69654 | 0.026541 | Down |
| P30086 | PEBP1 | 997375.5556 | 694735.5556 | 0.696564 | 0.030898 | Down |
| Q6YHK3 | CD109 | 4514400 | 3156611.111 | 0.699232 | 0.000461 | Down |
| Q9GZP4 | PITHD1 | 422788.8889 | 296756.6667 | 0.701903 | 0.009463 | Down |
| P04196 | HRG | 497906666.7 | 352323333.3 | 0.707609 | 0.01295 | Down |
| P08833 | IGFBP1 | 255348.8889 | 180806.6667 | 0.708077 | 0.037762 | Down |
| P30043 | BLVRB | 1720988.889 | 1227652.222 | 0.713341 | 0.007522 | Down |
| P13716 | ALAD | 2238033.333 | 1604773.333 | 0.717046 | 0.035356 | Down |
| Q13228 | SELENBP1 | 2077611.111 | 1494211.111 | 0.719197 | 0.003956 | Down |
| Q9NQ79 | CRTAC1 | 8992600 | 6504244.444 | 0.723289 | 0.023608 | Down |
| P00390 | GSR | 1262877.778 | 915178.8889 | 0.724677 | 0.000112 | Down |
| A0A140T902 | TNXB | 24702111.11 | 18423333.33 | 0.74582 | 0.001885 | Down |
| Q96S96 | PEBP4 | 771864.4444 | 575880 | 0.74609 | 0.034894 | Down |
| E9PF78 | ADAM22 | 85896.44444 | 64513.22222 | 0.751058 | 0.003019 | Down |
| P50395 | GDI2;GDI1 | 62343.55556 | 46913 | 0.752492 | 0.011792 | Down |
| P11166 | SLC2A1 | 263238.8889 | 199060 | 0.756195 | 0.011823 | Down |
| Q9Y2V2 | CARHSP1 | 40509.33333 | 30649 | 0.756591 | 0.027624 | Down |
| P35590 | TIE1 | 915490 | 695207.7778 | 0.759383 | 0.00396 | Down |
| Q15465 | SHH | 288860 | 219436.6667 | 0.759664 | 0.023568 | Down |
| P24666 | ACP1 | 305108.8889 | 233531.1111 | 0.765403 | 0.0188 | Down |
| O00602 | FCN1 | 398000 | 312487.7778 | 0.785145 | 0.014521 | Down |
| P28072 | PSMB6 | 370120 | 295083.3333 | 0.797264 | 0.034027 | Down |
| E9PHK0 | CLEC3B | 3588844.444 | 2861800 | 0.797415 | 0.003372 | Down |
| P21583 | KITLG | 127619.8889 | 102942.8889 | 0.806637 | 0.014169 | Down |
| Q12794 | HYAL1 | 843174.4444 | 680803.3333 | 0.807429 | 0.001651 | Down |
| Q09666 | AHNAK | 157180 | 127326.5556 | 0.810068 | 0.047213 | Down |
| O14818 | PSMA7 | 496288.8889 | 407232.2222 | 0.820555 | 0.036193 | Down |
| Q9Y646 | CPQ | 289752.2222 | 238726.6667 | 0.823899 | 0.009286 | Down |
| P61769 | B2M | 15752222.22 | 13040555.56 | 0.827855 | 0.013635 | Down |
| A0A0A0MSV6 | C1QB | 125150222.2 | 103902444.4 | 0.830222 | 0.017668 | Down |
| Q9UKU6 | TRHDE | 218836.6667 | 181766.6667 | 0.830604 | 0.049191 | Down |
| K7ES02 | BLMH | 1147565.556 | 956035.5556 | 0.833099 | 0.002426 | Down |
| Q9BUN1 | MENT | 6204522.222 | 7468255.556 | 1.203679 | 0.000913 | Up |
| G3V4U0 | FBLN5 | 675120 | 812660 | 1.203727 | 0.034521 | Up |
| P26842 | CD27 | 129218.8889 | 156332.2222 | 1.209825 | 0.018152 | Up |
| O00187 | MASP2 | 6016288.889 | 7304911.111 | 1.214189 | 0.00953 | Up |
| P0DP01 | IGHV1-3;IGHV1-69-2 | 310262.2222 | 384082.2222 | 1.237928 | 0.017007 | Up |
| Q14126 | DSG2 | 2342255.556 | 2904666.667 | 1.240115 | 0.004722 | Up |
| P02763 | ORM1 | 6562611.111 | 8189211.111 | 1.247859 | 0.013719 | Up |
| A0A096LPE2 | SAA2-SAA4;SAA4 | 62223666.67 | 77896777.78 | 1.251883 | 0.036115 | Up |
| A0A499FI48 | PDIA4 | 844764.4444 | 1062354.444 | 1.257575 | 2.95E-05 | Up |
| A0A0B4J1V0 | IGHV3-15 | 335104.4444 | 426493.3333 | 1.272718 | 0.036062 | Up |
| D6RE86 | CP | 1746744.444 | 2226166.667 | 1.274466 | 0.019462 | Up |
| F5GZZ9 | CD163 | 5566077.778 | 7118411.111 | 1.278892 | 0.001508 | Up |
| H0YJV3 | NID2 | 90369.77778 | 117201.6667 | 1.296912 | 0.004245 | Up |
| P01701 | IGLV1-51 | 1475488.889 | 1916000 | 1.298553 | 0.007922 | Up |
| A0A075B6I9 | IGLV7-46 | 536702.2222 | 700550 | 1.305286 | 0.027571 | Up |
| Q99832 | CCT7 | 83358.88889 | 109743 | 1.316512 | 0.000109 | Up |
| P00450 | CP | 1096693333 | 1459066667 | 1.330424 | 0.024528 | Up |
| P02750 | LRG1 | 87219333.33 | 116123222.2 | 1.331393 | 0.028794 | Up |
| Q8WUA8 | TSKU | 1578411.111 | 2115888.889 | 1.340518 | 0.010438 | Up |
| D6RF63 | KIAA1191 | 222651.1111 | 300136.6667 | 1.348013 | 6.41E-05 | Up |
| A0A1W2PP70 | HLA-DRA;HLA-DQA1;HLA-DQA2 | 190754.4444 | 258886.6667 | 1.357172 | 0.034221 | Up |
| Q14520 | HABP2 | 26571111.11 | 36113222.22 | 1.359116 | 0.01844 | Up |
| Q02383 | SEMG2 | 140461.1111 | 196924.4444 | 1.401986 | 0.007549 | Up |
| P01911 | HLA-DRB1;HLA-DPB1;HLA-DQB1;HLA-DRB5 | 130717.1111 | 184276.6667 | 1.409736 | 0.017745 | Up |
| P01877 | IGHA2 | 2026177.778 | 2951444.444 | 1.456656 | 0.016806 | Up |
| A0A0A0MS15 | IGHV3-49 | 319127.7778 | 470637.7778 | 1.474763 | 0.006955 | Up |
| P0DP08 | IGHV4-61 | 639780 | 977063.3333 | 1.527186 | 0.012736 | Up |
| Q8TDL5 | BPIFB1 | 114338.5556 | 179356.6667 | 1.568646 | 0.010388 | Up |
| Q9NYU1 | UGGT2 | 163658.8889 | 259853.3333 | 1.587774 | 3.72E-06 | Up |
| P01766 | IGHV3-13 | 296811.1111 | 472757.7778 | 1.59279 | 0.019592 | Up |
| P01780 | IGHV3-21 | 1864677.778 | 2971766.667 | 1.593716 | 0.034157 | Up |
| A0A7P0TAU9 | AGPS | 928575.5556 | 1490155.556 | 1.604776 | 0.00137 | Up |
| P01833 | PIGR | 253842.2222 | 408600 | 1.609661 | 0.003711 | Up |
| P06310 | IGKV2-30 | 597267.7778 | 961631.1111 | 1.61005 | 0.008303 | Up |
| A0A286YEY1 | IGHA1 | 10726622.22 | 17768966.67 | 1.65653 | 0.005137 | Up |
| P04279 | SEMG1 | 2862111.111 | 4747611.111 | 1.658779 | 3.33E-05 | Up |
| P0DOY3 | IGLC6 | 8174800 | 13825233.33 | 1.691201 | 0.022487 | Up |
| Q9NWV4 | C1orf123 | 1067096.667 | 1807944.444 | 1.694265 | 0.006475 | Up |
| P01009 | SERPINA1 | 37849000 | 64621444.44 | 1.707349 | 0.011551 | Up |
| P04275 | VWF | 39321222.22 | 67869666.67 | 1.726031 | 0.005892 | Up |
| D9J307 | HLA-B;HLA-C | 500628.8889 | 889362.2222 | 1.77649 | 0.017999 | Up |
| A0A075B6H9 | IGLV4-69 | 192525.5556 | 404558.8889 | 2.101326 | 0.022466 | Up |
| H3BTQ8 | SPINT1 | 671937.7778 | 1662776.667 | 2.474599 | 0.037218 | Up |
| I3L145 | SHBG | 12714444.44 | 33732000 | 2.653046 | 0.022351 | Up |
